# Variability in prey field structure drives inter-annual differences in prey encounter by a marine predator, the little penguin

**DOI:** 10.1098/rsos.220028

**Published:** 2022-09-14

**Authors:** Lachlan R. Phillips, Gemma Carroll, Ian Jonsen, Robert Harcourt, Andrew S. Brierley, Adam Wilkins, Martin Cox

**Affiliations:** ^1^ Macquarie University, Sydney, NSW, Australia; ^2^ School of Aquatic and Fisheries Sciences, University of Washington, WA, USA; ^3^ Resource Ecology and Fisheries Management Division, NOAA Alaska Fisheries Science Center, Seattle, WA USA; ^4^ Pelagic Ecology Research Group, Scottish Oceans Institute, Gatty Marine Laboratory, School of Biology, University of St. Andrews, St Andrews, Scotland KY16 8LB, UK; ^5^ Field Friendly, 203 Channel Highway, Kingston, Tasmania 7050, Australia; ^6^ Australian Antarctic Division, 203 Channel Highway, Kingston, Tasmania 7050, Australia

**Keywords:** prey field, active acoustics, predator-prey interactions, marine predator, foraging ecology, *Eudyptula minor*

## Abstract

Understanding how marine predators encounter prey across patchy landscapes remains challenging due to difficulties in measuring the three-dimensional structure of pelagic prey fields at scales relevant to animal movement. We measured at-sea behaviour of a central-place forager, the little penguin (*Eudyptula minor*), over 5 years (2015–2019) using GPS and dive loggers. We made contemporaneous measurements of the prey field within the penguins' foraging range via boat-based acoustic surveys. We developed a prey encounter index by comparing estimates of acoustic prey density encountered along actual penguin tracks to those encountered along simulated penguin tracks with the same characteristics as real tracks but that moved randomly through the prey field. In most years, penguin tracks encountered prey better than simulated random movements greater than 99% of the time, and penguin dive depths matched peaks in the vertical distribution of prey. However, when prey was unusually sparse and/or deep, penguins had worse than random prey encounter indices, exhibited dives that mismatched depth of maximum prey density, and females had abnormally low body mass (5.3% lower than average). Reductions in prey encounters owing to decreases in the density or accessibility of prey may ultimately lead to reduced fitness and population declines in central-place foraging marine predators.

## 1. Introduction

Marine predators search for food in an environment that is both patchy and highly dynamic [1]. In the open ocean, the distribution of prey biomass across the three-dimensional seascape, or ‘prey field’, has a complex, hierarchical structure [2]. At the finest spatial scale, prey species (e.g. small fish and zooplankton) form dense aggregations based on environmental cues [3] to improve locomotive efficiency [4], for predator avoidance [5], or for foraging and breeding [6]. These dense aggregations of prey are nested within patches of lower prey density that are separated by areas where prey is scarce [7,8]. This spatial organization, or ‘patchiness’, of the prey field varies over both horizontal and vertical dimensions in response to changing oceanographic conditions [9] and time of day, dynamically affecting both detectability and accessibility of prey to foraging predators [10]. Understanding variability in prey field structure and how it shapes the ability of marine predators to efficiently locate and catch prey is a central question in marine ecology [11].

Marine predators are expected to use foraging strategies that improve their chance of encountering aggregations of suitable prey across patchy landscapes [12]. Their movements should therefore track both prey field structure and its variability across space and time. Many studies have used the scale of predator foraging movements, and their dive characteristics and variability within foraging areas to infer prey field structures [13–15]. However, relatively little work directly links predator movements to prey field characteristics due to difficulties matching movements of wide-ranging predators at sea to information on prey distribution at relevant spatial and temporal scales (though see [2,8,16]). Most studies use indirect proxies of prey distribution such as sea surface temperature, or chlorophyll *a* concentration [17], but inferring spatial and temporal variability in biological activity from such data can produce mixed results [18].

Fisheries acoustic surveys enable direct sampling of prey densities at spatio-temporal resolutions relevant to marine predators [19,20]. Acoustic sampling has advantages over other sampling methods such as trawl surveys, as it provides information on the vertical distribution of prey [21,22] and can provide simultaneous qualitative and quantitative data on multiple components (e.g. prey species and size classes) of an ecosystem [19]. However, it remains difficult to relate acoustic estimates of prey field structure to marine predator foraging behaviour, as it requires *a priori* knowledge of where and when predators are expected to forage; a challenge with highly mobile marine vertebrates [23]. For this reason, predators such as seabirds with constrained foraging ranges during breeding may prove more tractable as prey field structure can be measured at spatial and temporal scales that are appropriately matched to the scales of predator foraging [8,24].

In southeast Australia, the East Australian Current (EAC) transports warm, nutrient-poor water from the tropics to temperate latitudes. Pulses of tropical water onto the shelf can be highly dynamic and play a major role in structuring spatial and temporal patterns of resource distribution across the coastal pelagic ecosystem [25–27]. Strong lines of evidence show that the EAC is intensifying, with a marked increase in the strength, duration, and frequency of southward incursions of EAC water [28–30] and seasonal dominance [31]. This intensification has consequences for prey availability to marine predators, including breeding seabirds that rely on their ability to efficiently track the distribution of prey within their home range to meet the energetic demands of breeding [8,32,33]. As a consequence, there is a strong need to further understand the relationship between prey distribution and marine predator foraging behaviour and success.

We measured the at-sea movements and dive behaviour of a central-place forager, the little penguin (*Eudyptula minor*), in southeast Australia over 5 years (2015–2019). While tracking foraging penguins, we used boat-based active acoustics to measure the density of acoustic backscatter as a proxy for the density distribution of prey within their foraging range. We aimed to determine whether penguin foraging behaviour reflects spatial variation in prey field structure across the seascape [11], and whether their prey encounter rate is robust to inter-annual variation in prey field characteristics. We hypothesized that the structure (distribution and density) of the prey field is a predictor of little penguin foraging behaviour, and that little penguins would adopt search strategies that increase their chance of encountering prey in this dynamic environment. We also hypothesized that the resulting differences in search strategies would lead to differences in penguin body weight (as a general proxy for foraging success). Our study provides insights into the functional relationship between the distribution of prey and the ability of predators to access it, and has implications for better understanding determinants of inter-annual variability in predator foraging behaviour and fitness in dynamic and changing ocean environments.

## 2. Material and methods

### 2.1. Penguin tracking

Penguins were tracked from a breeding colony on Montague Island (Baranguba) (36°15.11 S, 150° 13.60 E), 9 km off the southeast coast of New South Wales, Australia. Tracking was conducted during September and October over a 5-year period from 2015 to 2019. Penguins were tracked while brooding young chicks (0–4 weeks old) and therefore limited to making single-day foraging trips within approximately 25 km of the colony [34,35]. By targeting birds at this breeding stage, we were able to constrain the acoustic survey to a relatively small area around the colony and to focus on understanding patterns of foraging behaviour during the most energetically costly period of the penguins' annual cycle [36].

Penguins were caught in their nestboxes the night before they went to sea and equipped with biologgers to record GPS location and diving activity. In 2015–2016, a CatTrack GPS logging device (CatTrack, Pickens, SC, USA) modified with epoxy resin to withstand pressure at depth was attached. The loggers were inserted into waterproof heat-shrink tubing, then attached to feathers on the lower back with cloth tape (Tesa, Hamburg, Germany), positioned to reduce drag but not impede tail movement. Tags were 43 mm in length, 27 mm in width and 13 mm in height, and weighed 55 g in air and 17.4 g in seawater. Tags were programmed to record a location every 15 s in 2015 and every second in 2016. Dive depth loggers (G6a; CEFAS Technology Pty Ltd, Suffolk, UK) were attached immediately to the anterior of the GPS units on the middle back. Tags were 40 mm in length, 28 mm in width and 15 mm in height, and weighed 7.8 g in air and 2.3 g in seawater. The dive depth loggers recorded pressure in two modes: ‘shallow’ mode (less than 2 m: 1.5% of the full-scale pressure range) where pressure was recorded every 10 s, and ‘dive’ mode where pressure was recorded 30 times per second. ‘Dive’ mode was activated when depth was greater than 2 m and switched back to ‘shallow’ mode at less than 1.5 m. In 2017–2019, Axy-Trek loggers (Technosmart, Rome, Italy) that integrated both GPS and dive logging capabilities in a single unit were deployed. Axy-Trek tags were 36 mm in length, 23 mm in width and 12 mm in height, and weighed 57 g in air and 18 g in seawater. In 2017, we undertook three deployments of both Axy-Treks and CEFAS tags on the same individuals and the data were checked to ensure that the devices recorded equivalent data. Axy-Trek tags were attached to penguins in the same manner as the CatTrack tags and were programmed to record location and depth every second in 2017 and every 5 s in 2018–2019.

Penguins were weighed in a 100% cotton bag using a spring balance scale (Pesola, AG Switzerland) at device attachment and removal. Penguins were handled for less than 10 min during logger attachment and removal. As penguins were fasting prior to foraging trips while their partner was at sea, penguin weight anomalies were calculated from the weight recorded at the time of logger deployment and compared between years using generalized linear models fit separately to females and males. Animal research protocols were approved by the Macquarie University Animal Ethics Committee (Animal Research Authority 2014/057-17).

### 2.2. Acoustic data collection and processing

**Figure 1.**
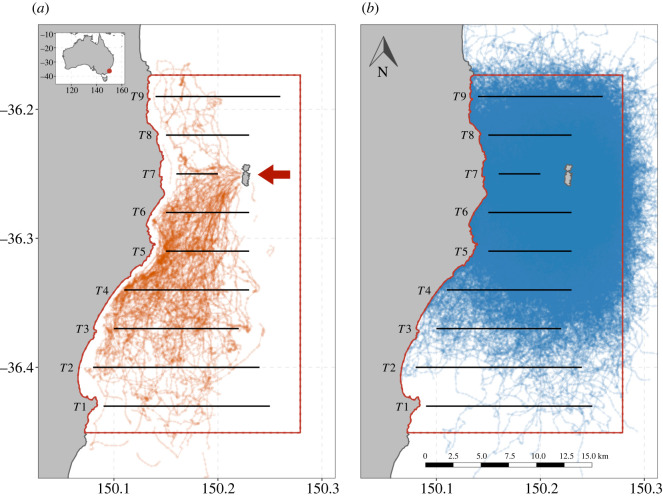
Map of the study area showing the nine acoustic transects (black lines) and the prey field kriging boundary (red border) overlaid over the (*a*) 109 penguin tracks and (*b*) the 3117 simulated tracks. The red arrow indicates the location of Montague Island.

Boat-based acoustic surveys were conducted within the same time window as the penguin logger deployments, coinciding with the peak of the penguin breeding season (table 1). Surveys were undertaken during daylight hours (when penguins forage) from a 6 m rigid-hulled inflatable vessel, R/V Pelagica, travelling at 4–6 knots along cross-shelf transects spaced 3.5 km apart and ranging in length from 7 to 14 km (figure 1). In 2015, seven transects were sampled and an additional two transects were added to the northern survey area in 2016–2019, making a total survey extent of 31.5 km north to south along the continental shelf (figure 1). An EK80 (Simrad, Horten, Norway) scientific echosounder operating a 70 kHz transducer (3 dB beamwidth 18°, transmit power 280 W, pulse duration 1.024 ms, 2 Hz pulse repetition rate) in continuous wave mode was mounted on a pole connected to the vessel via a retractable arm (transducer depth = 0.75 m). Calibrations were performed each survey year following the procedure of Demer *et al*. [37].

**Table 1.** Little penguin deployment information and mean trip statistics for each year. Estimated locations occur at regular 5 min intervals. Recorded dives were all dives greater than 2 m depth and longer than 5 s.

	2015	2016	2017	2018	2019
tracks	31	46	14	14	11
individuals	30	14	14	12	8
sex (F%)	53%	50%	43%	58%	38%
observed locations	16 472	50 067	242 233	32 679	14 576
estimated locations	5076	5560	2126	1970	1566
logger acquisition interval (seconds)	15	1	1	5	5
mean trip duration (hours)	12.7 (±1.9)	10.0 (±0.8)	12.6 (±2.7)	11.5 (±2.6)	11.5 (±2.5)
mean max distance from colony (km)	16.0 (±3.9)	14.0 (±2.4)	16.5 (±3.4)	14.0 (±3.7)	15.3 (±5.0)
mean trip distance travelled (km)	25.2 (±6.0)	35.1 (±7.6)	30.1 (±7.5)	30.7 (±6.6)	25.7 (±9.9)
recorded dives	10 688	4651	4145	2385	1443
mean distance from coast of foraging locations	1.72 ± 1.33 km	3.15 ± 2.44 km	6.35 ± 1.79 km	4.03 ± 1.60 km	4.21 ± 2.71 km
penguin tracking period	30 Sept–8 Oct	22 Oct–31 Oct	4 Oct–13 Oct	30 Sept–7 Oct	27 Sept–9 Oct
acoustic survey period	30 Sept–7 Oct	22 Oct–2 Nov	2 Oct–13 Oct	27 Sept–1 Oct	30 Sept–2 Oct
logger type	CatTrack/CEFAS	CatTrack/CEFAS	Axy-Trek	Axy-Trek	Axy-Trek

Acoustic data were processed using Echoview version 11 (Echoview, Hobart, Australia). Seabed returns and surface noise were removed from the acoustic data prior to aggregation detection. Aggregations were isolated from the acoustic record by applying the SHAPES edge detection algorithm [38,39] implemented in the ‘Schools Detection’ module of Echoview to a 3 × 3 identity matrix convolution of the acoustic data. The minimum detection threshold was −65 dB re 1 m^−1^, and schools detection algorithm parameters were as given in Carroll *et al*. [8] (electronic supplementary material, table S2). Mean volume backscattering strength (*S*_v_; see MacLennan *et al*. for definition [40]) was calculated using a −80 dB re 1 m^−1^ minimum data threshold.

Due to logistical challenges of working from a small boat, net tows could not be performed in tandem with acoustic surveys to sample species composition and length frequency of fish schools or other targets. Penguin-borne video cameras deployed during the period of acoustic surveys in 2018 and 2019 showed that penguins were feeding primarily on small schooling pelagic fish near the surface (Phillips *et al*. unpublished data). A diet study on greater crested terns breeding on Montague Island that overlapped the acoustic survey in 2018 showed a predominance of Australian anchovy (*Engraulis australis*), barracouta (*Thyrsites atun*), and trevally (*Pseudocaranx* spp.) being brought back to the colony [33]. Line fishing with baited hooks over acoustically observed fish schools in 2018 indicated that schools were commonly composed of small pelagic species such as horse mackerel (*Trachurus declivis*) and blue mackerel (*Scomber australasicus*), and in 2019 large swarms of krill were observed from the boat during acoustic surveys. However, despite these lines of evidence that shallow aggregations observed in this region are likely to be composed primarily of coastal pelagic fish species that are suitable prey for penguins (with occasional observations of krill), unavoidable uncertainty remains as to the species composition and size structure of acoustically detected schools. Further, we assume that the conditions observed during acoustic surveys adequately reflect patterns of inter-annual variability in prey field structure.

We describe prey field structure as a combination of ‘acoustic density’ and depth. We use the term ‘acoustic density’ to refer to the mean volume backscattering strength (*S*_v_), a measure of the intensity of sound returned from objects in the water column that is proportional to the log density of animals [40]. To create three-dimensional representations of prey field structure, we created three-dimensional maps of acoustic density using three-dimensional multi-Gaussian ordinary kriging for each year. This involved applying a normal-score transformation to the acoustic data (*S*_v_) with the final interpolated estimates of acoustic density being back-transformed to log space [41,42]. Variogram models (with nugget, Gaussian, and cubic structures) were fit to the empirical variograms to describe spatial autocorrelation in the acoustic data. Variogram models were used to krige the data onto a 200 × 200 × 1 m resolution grid (*RGeoStats* package version 11.2.12) encompassing an area from the coast to the transect margins plus a buffer of 1.25 km (figure 1). Kriging was performed using a continuous moving search neighbourhood with a horizontal search radius of 50 km and vertical search radius of 20 m using a minimum of 3 and a maximum of 10 neighbours. To prevent a discontinuity of the kriging map, a penalization method was applied to the outermost data points of the neighbourhood using the approach outlined by Rivoirard & Romary [43].

### 2.3. Track processing and simulations

To understand how prey field structure influences inter-annual variability in prey encounters, we compared rates of acoustic density encountered by real penguins moving through the kriged prey field, with encounter rates by simulated penguins moving at random through the same kriged prey field in each year.

#### 2.3.1. Penguin track processing

Penguin tracks were processed to remove foraging trips that were longer than a single day, and erroneous locations were removed by recursively filtering points where calculated swimming speeds were greater than 20 m s^−1^. To account for track variability in sampling frequencies and time spent underwater, surface locations were estimated at 5-min intervals using a continuous-time correlated random walk model in the R package *crawl* version 2.2.1 [44]. The model was fit in state-space form using a Kalman filter [45]. As *crawl* is optimized for use with ARGOS data, we fixed observed location standard errors at 5 m to account for the negligible error of GPS devices [46].

#### 2.3.2. Simulated penguins

A two-state hidden Markov model (HMM) fit with the R package *momentuHMM* version 1.5.1 [47] was used to classify the regularized penguin tracks into ‘foraging’ and ‘transiting’ behaviour states. The ‘foraging’ state reflects periods of area-restricted search characterized by short step lengths and a wide distribution of turning angles, and the ‘transiting’ state is characterized by straight, directed travel, with a narrow distribution of turning angles centred around zero. Step length distributions were estimated using a gamma distribution, and turning angle distributions were estimated using a von Mises distribution. Core foraging ranges by penguins in each year were calculated using kernel utilization distributions (75% UD contours) of locations classified by the HMM as foraging, using the R package *MASS* version 7.3–53.1 and the bandwidth parameter *h* set to the normal reference distribution [48]. The mean distance from the coast of foraging locations was also calculated (table 1).

We simulated penguin tracks to determine whether penguins' prey encounters were better than random. Simulated penguin tracks were generated using similar movement characteristics to real penguins, but otherwise moving randomly through the study area. Movements were simulated from the step length and turning angle parameters estimated by the two-state HMM. To avoid biasing the simulations to years with more penguin tracks, HMM's were fit to a random subset of 10 penguin tracks from each year (*N* = 50). Five thousand tracks were simulated from the HMM at the same 5 min interval as the penguin tracks and with same number of total locations, using a modified version of the ‘simData’ function in *momentuHMM* to prevent location fixes being simulated over land. To ensure spatial overlap between track data and the kriging models, any real or simulated tracks that had more than 20% of location fixes outside of the survey area were removed from the analysis. A total of 3117 simulated tracks were kept after filtering for tracks that left the survey area (figure 1).

### 2.4. Prey encounter index

To explore how prey encounter rates of real penguins compared to the simulations, we calculated an index of prey encounter for each individual real and simulated track by running the tracks through the kriged acoustic fields. ‘Prey encounter’ was defined as the average amount of linear acoustic density encountered per hour of foraging across each track (*S*_v_/hr). Because simulated tracks did not have associated dive profiles, we incorporated the depth of prey distribution into the prey encounter index by taking the mean of kriged acoustic density across all depth bins and weighted by the frequency distribution of penguin maximum dive depths for that year to create a two-dimensional version of the kriged acoustic fields.

A bootstrapping method was used to compare prey encounter indices between real and simulated tracks, providing robust between-group comparisons that do not rely on assumptions made by statistical tests. A sample of simulated tracks equal to the number of real tracks for each year was drawn at random 100 000 times. The mean prey encounter rate between the real tracks and the sample of simulated tracks was then compared in each draw to determine whether the real penguins were encountering more prey than would be achieved by random movement. Prey encounter performance was then calculated by subtracting the mean simulated prey encounter rate from the mean real prey encounter rate (*S*_v_ mean anomaly). The two most northerly transects were not acoustically sampled in 2015, so simulated 2015 tracks were filtered to only use tracks that remained south of 36°14.1 S and avoid extrapolating beyond the range of available acoustic survey data.

### 2.5. Dive behaviour and vertical prey distribution

To determine how penguin dive behaviour related to the depth distribution of acoustic prey density, the maximum depth of each dive, and the frequency of maximum dive depths across the entire study area and within the core foraging ranges (inside the 75% UD) were assessed for each year. This was visually compared to the vertical distribution of mean prey density across all cells for each 1 m kriged depth bin for the entire study area and within the core foraging ranges. To focus primarily on foraging behaviour, short, shallow dives less than 2 m and/or less than 5 s associated with transiting were removed before analysis.

All analyses were performed in the R programming language version 3.6.3 [49], and all values are means ± s.e. unless otherwise stated.

## 3. Results

**Figure 2.**
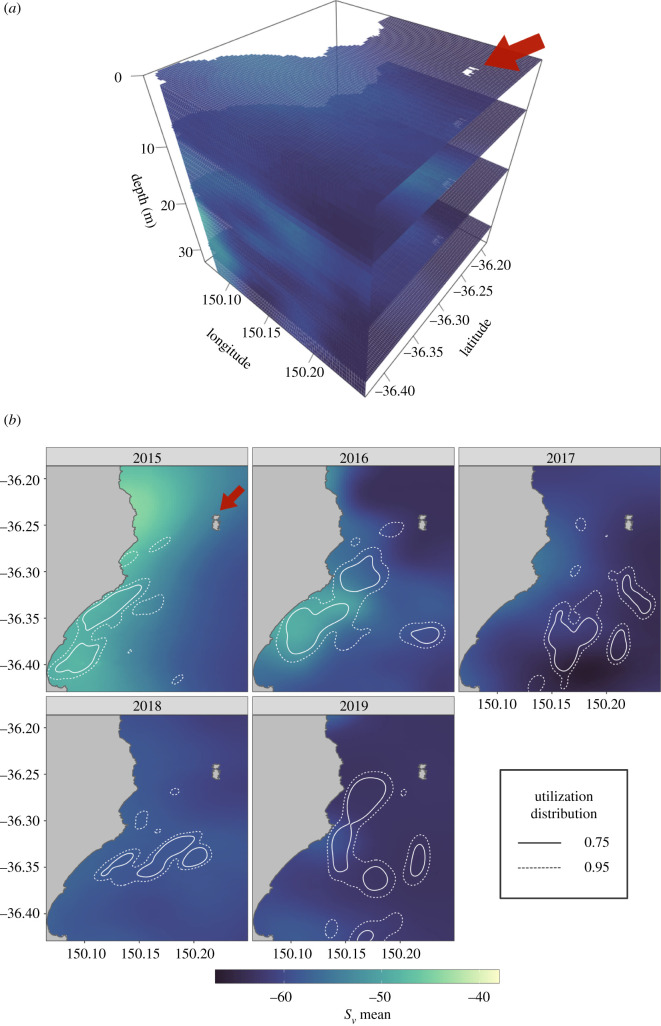
(*a*) An example of the three-dimensional kriging of acoustic density (*S*_v_ mean) for the top 30 m of the water column for 2019. (*b*) Mean acoustic density (*S*_v_ mean) present in the top 30 m of the water column for each year weighted by the frequency distribution of penguin maximum dive depths. The kernel utilization distributions of real penguin GPS locations classified by a HMM as ‘foraging’ behaviour are overlaid as white contour lines. The red arrow indicates the location of Montague Island.

Data from 116 penguin foraging trips by 78 individuals during acoustic survey periods between 2015 and 2019 were included in analyses after removing tracks that left the survey area or had insufficient data quality (table 1). This represented 16 298 location estimates after regularizing the tracks to 5 min intervals. On average, foraging trips were 11.4 (±2.1) h long and covered a total distance of 31.1 (± 7.9) km, with a mean maximum displacement from the colony of 15.3 (±3.9) km (table 1). Foraging behaviour was concentrated close to the coast southwest of Montague Island in all years except 2017 when foraging took place farther offshore (table 1 and figure 2). The 31 208 penguin dives were generally short and shallow, with 62% of dive activity occurring within the top 10 m of the water column and greater than 99% within the top 30 m.

**Figure 3.**
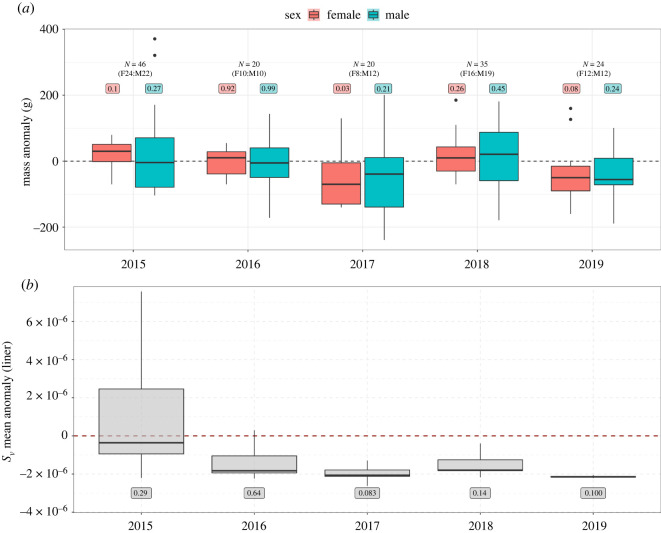
(*a*) Boxplots of little penguin body mass anomaly (g) for females and males, and (*b*) *S*_v_ mean anomaly values transformed to linear scale for each cell of the three-dimensional kriging models up to 30 m depth for each survey year. The labels above and below the boxplots indicate the *p*-values for each year compared to the overall mean for all years using (*a*) generalized linear models fit separately to females and males, and (*b*) Wilcoxon signed-rank tests. The generalized linear models in (*a*) were fit by forcing the intercepts = 0, and overall means were calculated separately for females and males.

Three-dimensional maps of acoustic density (*S*_v_; dB re 1 m^−1^) representing a proxy for prey density showed inter-annual variability (figure 2). Acoustic density estimates were highest in 2015 and lower in 2017 and 2019 (figure 3). There was more variability in the magnitude of acoustic density than in the location of high-density areas, with the highest acoustic density concentrated within approximately 5 km of the coast in all years (figure 2). Core foraging areas used by penguins overlapped these inshore regions of relatively high acoustic density most clearly in 2015 and 2016, when there were dense patches of acoustic energy located closer to the coast (figure 2).

**Figure 4.**
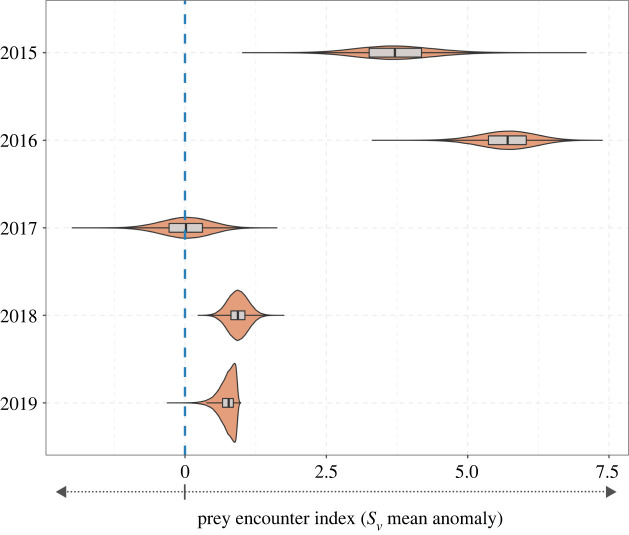
The distribution of mean prey encounter index (*S*_v_ mean) of the penguin tracks minus the mean prey encounter index of an equal-sized subsample of the simulated tracks randomly drawn 100 000 times for each of the survey years. Positive values indicate that the identified prey encounter index was greater than what would be expected from random foraging (blue dotted line).

Penguins had greater prey encounter indices than the simulated penguin tracks, outperforming random simulations greater than 99% of the time in all years except 2017. In 2017, real penguin performance was approximately equivalent to the random simulations, with real penguins performing better than random only 47% of the time (figure 4). Additionally, real penguin performance in 2015 and 2016 significantly outperformed the simulations (3.73 ± 0.68 and 5.70 ± 0.49 *S*_v_ mean anomaly, respectively), while 2018 and 2019 had *S*_v_ mean anomaly values of 0.94 ± 0.18 and 0.74 ± 0.15, respectively.

**Figure 5.**
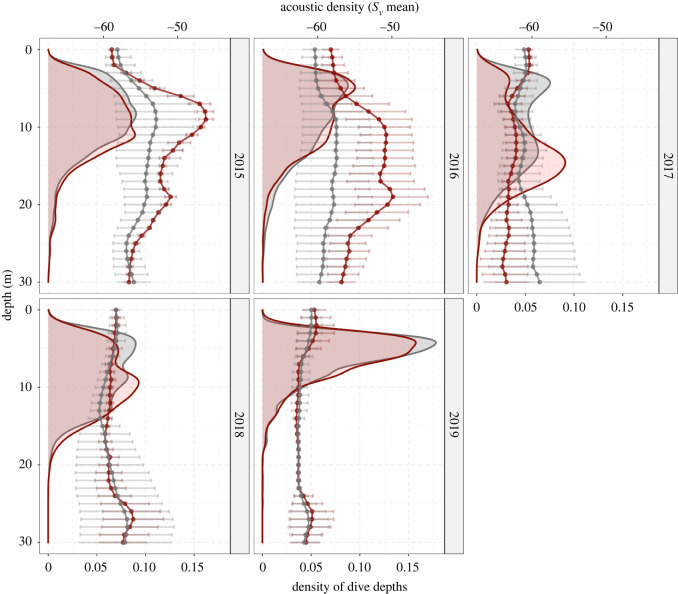
The distributions of little penguin maximum dive depth across the entire survey area (grey-shaded area) and within the foraging hotspots (dives within the 75% utilization distribution of foraging locations) (red-shaded area), and the respective mean vertical distribution of acoustic density (*S*_v_ mean) to 30 m depth (dotted lines) over the entire survey area (grey) and within the foraging hotspots (red) for each of the surveyed years. Error bars show the s.d. from the mean.

Penguin diving behaviour matched the vertical distribution of acoustic density in all years at depths most accessible to penguins (figure 5). Maxima in the frequency of dive depths showed similar patterns to the maxima of acoustic density at depths less than 20 m. Penguins rarely dived to depths greater than 20 m regardless of the levels of acoustic density below this depth. In 2017, for example, the distribution of acoustic density was both sparse and deep, with the greatest levels of acoustic density located at depths greater than 20 m. In 2019, the distribution of acoustic density was similarly sparse, but concentrated within the top 5 m of the water column, and penguin diving behaviour was focused at this depth. Diving behaviour within the core foraging ranges (inside the 75% kernel density estimate of foraging locations) was similar to diving behaviour outside this area, except in 2017 when penguins were favouring deeper dives (figure 5). In most years, the vertical distribution of acoustic density (*S*_v_ mean) was higher within the core foraging ranges, except in 2017 when it was lower and 2019 when there was no difference (figure 5).

Mean penguin body mass was variable between years (figure 3*a*), with females showing the highest inter-annual variability. Female body mass was 5.3% lower than average in 2017 (*p* < 0.05, GLM fit to annual mass anomalies).

## 4. Discussion

We hypothesized that foraging movements of little penguins would track the structure of the prey field, and that penguins would vary their behaviour to maximize their chance of encountering prey under diverse foraging conditions. We investigated this by (i) comparing an index of prey encounters by penguins with prey encountered by randomly foraging (simulated) penguins and (ii) comparing penguin dive depths with the vertical distribution of acoustic density (as a proxy for density distribution of prey). We found that penguins use movement patterns that provide a higher rate of prey encounter than would be achieved by making random movements through their foraging range. However, in years when prey was abnormally sparse and deep, penguin prey encounters appeared close to random and there was evidence of a reduction in body mass in those years. Our finding that inter-annual variability in penguin prey encounter rates mirrored the variability of prey field structure suggests that changes in prey abundance and accessibility are important determinants of penguin foraging success, which may have costs for individual fitness.

The distribution of penguin diving depth closely matched the vertical distribution of prey in each of the survey years within the range of depths the penguins could access, particularly in years when acoustic density was higher such as 2015–2016 (figure 5). Diving effort was concentrated at depths less than 20 m in all years, even though the peaks in depth distribution of acoustic density in 2017, 2018, and 2019 were at depths greater than 20 m. This suggests that a penguin's ability to detect prey from the surface may be limited to certain depths of the water column and/or a trade-off exists between the potential energetic gains of prey and the energy required to access them, considering the costs associated with diving [50]. Greater diving effort by little penguins as measured by diving time and depth is associated with lower fledging success [51], suggesting that while penguins can access prey at greater depths than we observed (with dives as deep as 67 m recorded) [50], they do so at the cost of increased energy expenditure and ultimately reductions in reproductive fitness. This supports previous work showing that prey shallowness and aggregation density increase prey encounter and consumption rates [8,52].

An animal's foraging efficiency is directly influenced by its ability to effectively locate prey [53]. In this study, penguins had a greater prey encounter index than random tracks in greater than 99% of cases for 4 out of 5 years. In 2015 and 2016, penguin foraging performance was considerably better than random, with penguins outperforming the simulated tracks 100% of the time (figure 4). These high prey encounter rates can be partly explained by the fact that a majority of acoustic energy accessible to penguins (less than 20 m) was concentrated close to the coast in these years, creating an inshore ‘hotspot’ of prey density that penguins targeted during foraging (figure 2). Notably in these years, acoustic density was highest within the core foraging ranges, particularly at depths little penguins were targeting (figure 5). In 2018 and 2019, shallow acoustic density was both lower and distributed more widely, resulting in a more homogeneous prey field and lower spatial overlap with penguin foraging locations. Additionally, the vertical distribution of acoustic density inside penguin core foraging ranges in these years was equivalent to the distribution across the entire study area (figure 5). In a completely uniform environment, there is no advantage to any foraging strategy compared with random movements, and this variation in the heterogeneity of prey distribution appears to play a major role in the detectability of prey across the foraging range and the ability of penguins to move between neighbouring schools [8].

In 2017, penguins’ performance was close to random, only outperforming simulated tracks in 47% of cases, and female body mass was also 5.3% lower than average. Additionally, the vertical distribution of acoustic density in the penguins' core foraging areas was lower when compared with the entire study area (figure 5). Consequently, penguins’ ability to detect prey may be compromised below certain prey densities. However, prey density was also lower than average in 2019, when penguins performed better than simulated tracks greater than 99% of the time. While mean acoustic density was similar in these two years, the vertical structure of prey density was different. In 2019, the highest levels of acoustic density were near the surface, and penguin diving behaviour was characterized by short and very shallow dives of approximately 5 m depth. Conversely in 2017, penguin diving behaviour showed a bimodal distribution, with penguins primarily making shallow dives to approximately 5 m but typically diving to deeper depths (approx. 15 m) within core foraging areas (figure 5). Because penguins in 2019 were primarily targeting prey located very near the surface, this may have made it easier for them to efficiently locate and track prey species despite relatively low levels of prey density. This may explain their greater prey encounter rates over random searching compared with 2017. Interestingly, this did not appear to translate into increased body condition as measured by penguin body weight (figure 3*a*), despite improved prey encounters and more accessible prey. This suggests that while the three-dimensional distribution of prey can be an important factor in determining prey encounters, it may not always translate into foraging success as this is also influenced by other factors such as absolute prey density and the quality of prey available within the foraging range. Female body mass was 5.3% lower than average in 2017 (*p* < 0.05, GLM fit to annual mass anomalies). This indicates that females may be more sensitive to these changes in prey availability, possibly due to the additional energetic demands of egg production [36].

Few studies have had the opportunity to match movements of wide-ranging predators at sea to information on prey distribution due to the logistical challenges associated with collecting this data simultaneously at relevant scales. Because of these challenges, it is important to acknowledge that we have made the assumption that our ‘snapshots’ of prey field distribution are representative. If this assumption is not true, then some of the patterns in prey field structure observed in this study may be attributed to this mismatch. Because the scales of spatio-temporal variability of prey distribution in coastal systems is still an outstanding question, this is a common limitation of these kinds of studies [2,8,10,11,16].

The southeast Australian region is an extremely dynamic environment as a result of the influence of the highly variable EAC extension [54], which has intensified over the past few decades [28,31]. This intensification of the EAC may significantly impact factors that influence ecosystem productivity and prey field structure in southeast Australian waters. Significant change is already evident in the region, including changes to nutrient loading regimes [55], increased frequency and persistence of prolonged marine heatwaves [56], changes in the timing of seasonal events [26,31], changes in planktonic species assemblages and abundances [57], and shifts in the distributions of pelagic sharks and fishes [58–60]. The complex and interactive relationship between these factors and prey distribution makes it difficult to accurately predict the impact these changes may have on little penguin foraging behaviour and reproductive success. More work is needed to understand variability in prey field structure over multiple spatial and temporal scales in relation to ocean climate, and to understand how effectively predators can respond to changes at these scales. While marine predators like little penguins may be able to modify their behaviour and so adjust to environmental change in the short term [13,14], poor performance in years of low prey availability points to potential limits in their adaptability under adverse environmental conditions.

## Data Availability

All data and code used in this study are available from the Zenodo Digital Repository: https://doi.org/10.5281/zenodo.4671120 [61].
